# Groundwater contamination in Ibadan, South-West Nigeria

**DOI:** 10.1186/2193-1801-3-448

**Published:** 2014-08-20

**Authors:** Christiana Ndidi Egbinola, Amobichukwu Chukwudi Amanambu

**Affiliations:** Department of Geography, University of Ibadan, Ibadan, 23402 Nigeria

**Keywords:** Groundwater, Shallow aquifers, Geogenic contaminants, Arsenic, Fluoride, Awareness

## Abstract

Groundwater is the main source of water for domestic use in Nigeria because it is perceived to be clean. The presence of geogenic contaminants (arsenic and fluoride), and the level of awareness of their presence in groundwater in Ibadan, Nigeria was examined in this study. A total of one hundred and twenty groundwater samples were collected from hand dug wells which tap into shallow aquifers and their location taken with the aid of a GPS. The concentration of arsenic was determined by Atomic Absorption Spectrophotometry (AAS) while concentration of fluoride was determined by single beam spectrophotometer. Three hundred and fifty semi structured questionnaires were also administered within the study area to determine the level of awareness of contamination problem. Simple summary statistics including mean (m) standard deviation (s) and minimum-maximum values of the hydro-chemical data was used in the data analyses, while spatial concentrations were mapped using ArcGIS. The results showed arsenic concentration exceeding the WHO (2011) recommended concentration for drinking water in 98% and 100% of the dry and wet season samples. Concentration of Fluoride exceeded the recommended limits in 13% and 100% of the dry and wet season samples. Questionnaire analyses revealed that 85% of respondents have never tested their wells, 55% have no knowledge of geogenic contamination, while 92% never heard of arsenic or fluoride (52%). The study recommends enlightenment on geogenic contamination and testing of wells for remediation purposes.

## Background

Access to safe drinking water is essential to health, it is a basic human right (WHO, 2011). Therefore, an adequate and safe supply of water is essential for development. The World Health Organization’s 2002 estimates showed that more people die each year from the consequences of unsafe or inadequate water supplies than from all forms of violence (WHO, 2002). Groundwater has become an indispensable source of drinking water worldwide and especially in developing countries. The 2006 Nigerian household population census revealed that 49.4% of sampled households depend on groundwater as the main source of water for domestic use. This high dependence stems from the fact that groundwater is thought to be free of the pathogens widely found in surface waters (Eawag, 2011). However, groundwater may contain a wide variety of dissolved inorganic chemical constituents resulting from interactions between water and geologic materials. These geogenic contaminants can have a negative effect on human health.

Geogenic contaminants affect the health of hundreds of millions of people worldwide, it is estimated that around 200 million people worldwide are affected by arsenic and fluoride contamination alone, roughly 5 per cent of all those who use groundwater for drinking (Amini *et al.*, 2009). The health implications of the ingestion of arsenic contaminated drinking water include dermal lesions such as hyperpigmentation and hypopigmentation, peripheral neuropathy, skin cancer, bladder, lung and kidney cancers and peripheral vascular diseases (IPCS; Amini *et al.*, 2009; WHO, 2011). The ingestion of elevated concentrations of fluoride leads to dental and skeletal fluorosis (Edmunds and Smedley, 2005; IPCS; WHO, 2011).

In spite of the health risks associated with the ingestion of groundwater with elevated concentration of As. and F., few studies have been conducted to determine the scale of As and F contamination of groundwater in Nigeria and even fewer studies on users awareness of geogenic contamination. The knowledge of areas with elevated geogenic contaminants in Nigeria is of critical importance in safeguarding the health of the citizens, since the majority of people (especially the urban and rural poor) depend on untreated groundwater for domestic purposes. This study therefore aims at analysing the concentration of arsenic and fluoride in groundwater from hand-dug wells in the Ibadan region so as to reveal areas (if any) with elevated concentration of arsenic or fluoride. In addition, the study will determine the level of awareness of geogenic contamination among well owners and users within the study area.

## Methods

**Figure 1:**
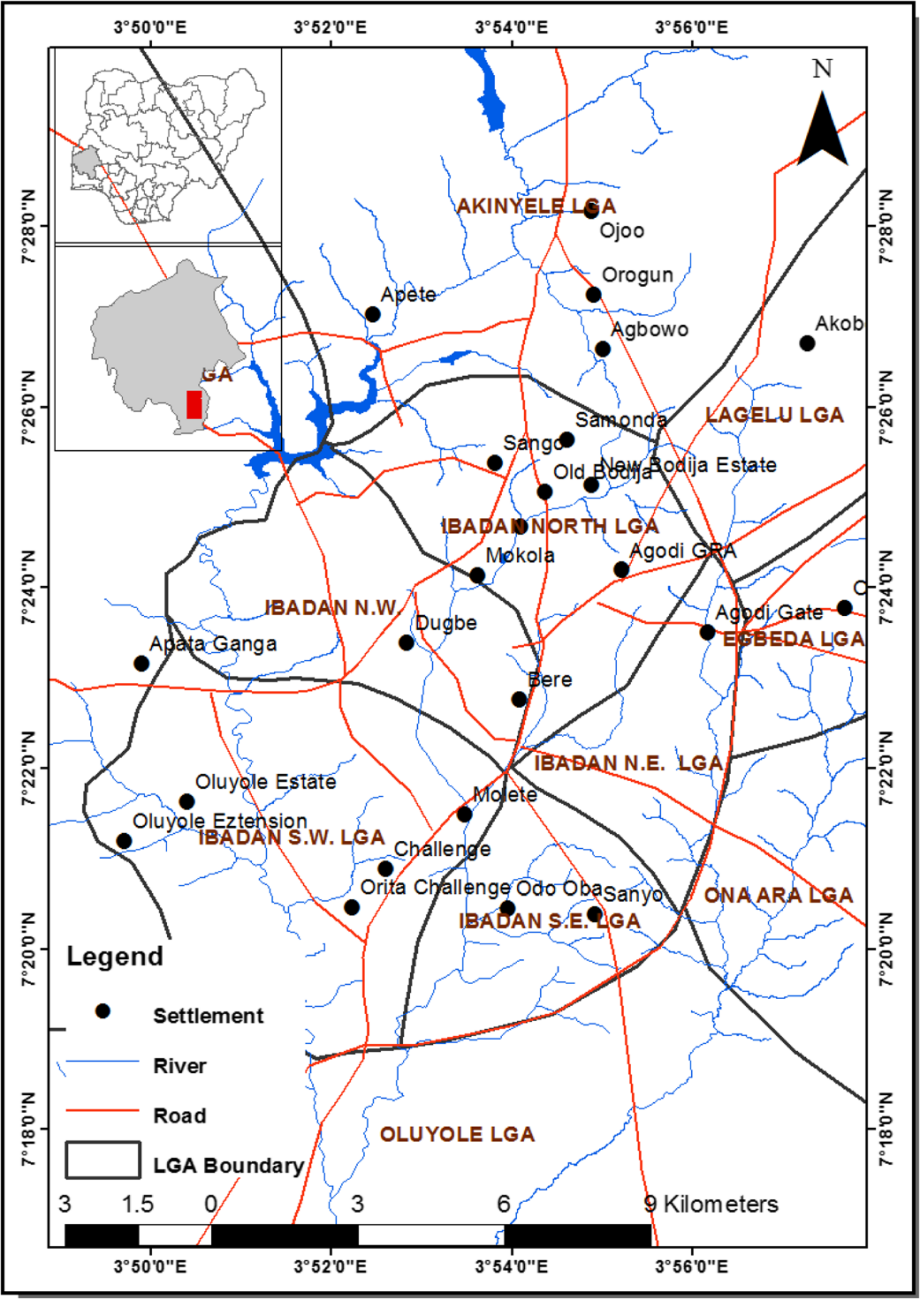
**The study area.**

The study was carried out in Ibadan the capital of Oyo state, located between latitude 7° 02’ 49" and 7° 43’ 21" N longitude 3° 31’ 58" and 4° 08’ 20" E (see Figure 1). Mean annual rainfall is about 1,205 mm, mean temperature is 28°C, ranging between 18°C and 37°C, while relative humidity is high all year round at about 74.55%. The study area is underlain by metamorphic Pre-Cambrian Basement Complex rocks with Gneisses as the predominant rock type. The Basement Complex rocks are generally considered as poor aquifers because of their low porosity and permeability (Davis and De Wiest 1966). Availability of groundwater depends therefore on the depth of the weathered material (overburden) and the presence of joints and fractures in the rock.

A total of one hundred and twenty groundwater samples from sixty wells that tap into shallow aquifers were collected while 350 questionnaires were administered within the study area. Groundwater sampling was carried out in October and November 2012 at the end of the rainy season and during March and April 2014 at the start of the rainy season, while questionnaire administration was carried out in February 2013.

Groundwater samples were collected in prewashed polyethylene bottles (750 ml) and GPS reading recorded for each sampling point. Arsenic concentration was tested using AAS (Buck Scientific Atomic Absorption Spectrophotometer), 210/211 VGP, Spectrophotometer and the Beckman DU-7500 single beam spectrophotometer while the Hitachi U-1500 UV/Vis single beam spectrophotometer was used to determine fluoride concentration. These analyses were carried out in the Agronomy laboratory, University of Ibadan and the WATSAN laboratory, Ibadan.

Three hundred and fifty owners and users of well responded to the questionnaire interviews. The respondents were selected randomly in different communities within the study area including Agbowo, Orogun, Barika, Samonda, Songo, Bodija, Agodi, Mokola, Dugbe, Oluyole, Challenge, Apata, Apete, Ojoo, Beere and Iwo road.

Simple summary statistics including mean (m) standard deviation (s) and minimum-maximum values of the hydro-chemical data were used in the data analyses. The concentration of contaminants was mapped with the aid of Arc GIS software.

## Results

### Arsenic and fluoride concentration in groundwater

Table 1 shows the concentrations obtained for As. and F in water samples during the rainy and dry seasons in the various locations sampled.

**Table 1:** **Summary of physico-chemical analysis of water samples**

	N	Min	Max	Mean	Std. Dev.	Variance	Skewness	Kurtosis
Arsenic (Dry)	60	0.00	0.38	0.14	0.11	0.01	0.82	-0.21
Arsenic (Rainy)	60	1.03	3.06	2.27	0.62	0.38	-0.50	-0.86
Fluoride (Dry)	60	0.01	3.70	0.56	0.84	0.70	2.18	4.99
Fluoride (Rainy)	60	1.82	6.38	3.37	1.22	1.48	0.89	-0.33

Source: Fieldwork, 2012/2014.

The concentration of arsenic (As) in groundwater samples collected at the end of the rainy season ranged from 0.00 mg/l to 0.38 mg/l with a mean of 0.14 mg/l, while fluoride concentration ranged between 0.01 mg/l and 3.70 mg/l with a mean of 0.56 mg/l. Concentration levels of arsenic for the samples collected at the beginning of the rainy season ranged between 1.03 mg/l and 3.06 mg/l with a mean of 2.24 mg/l while concentration levels for fluoride (F) ranged between 1.82 mg/l and 6.38 mg/l with a mean of 3.37 mg/l.

**Figure 2:**
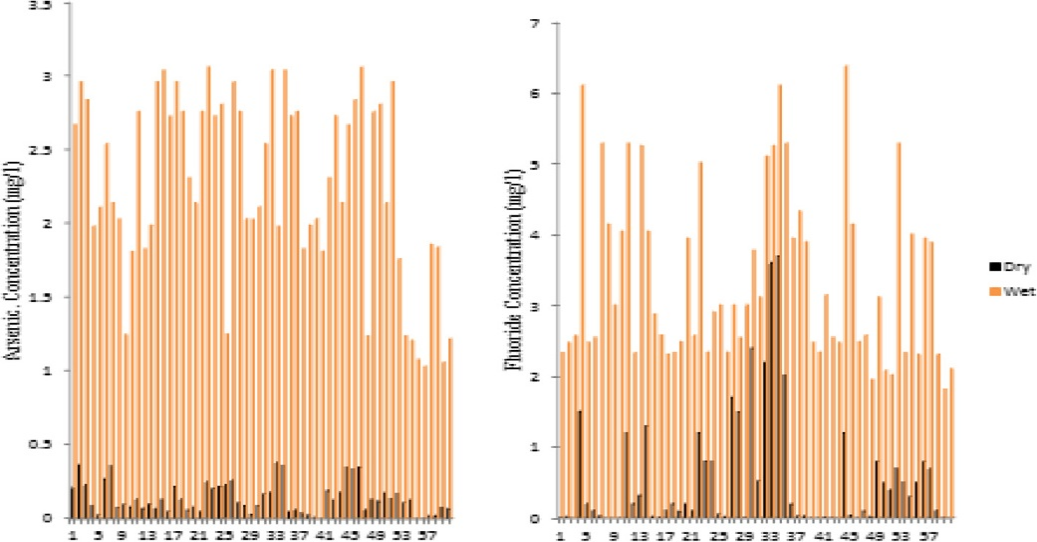
**Arsenic and fluoride concentration in studied wells.**

**Figure 3:**
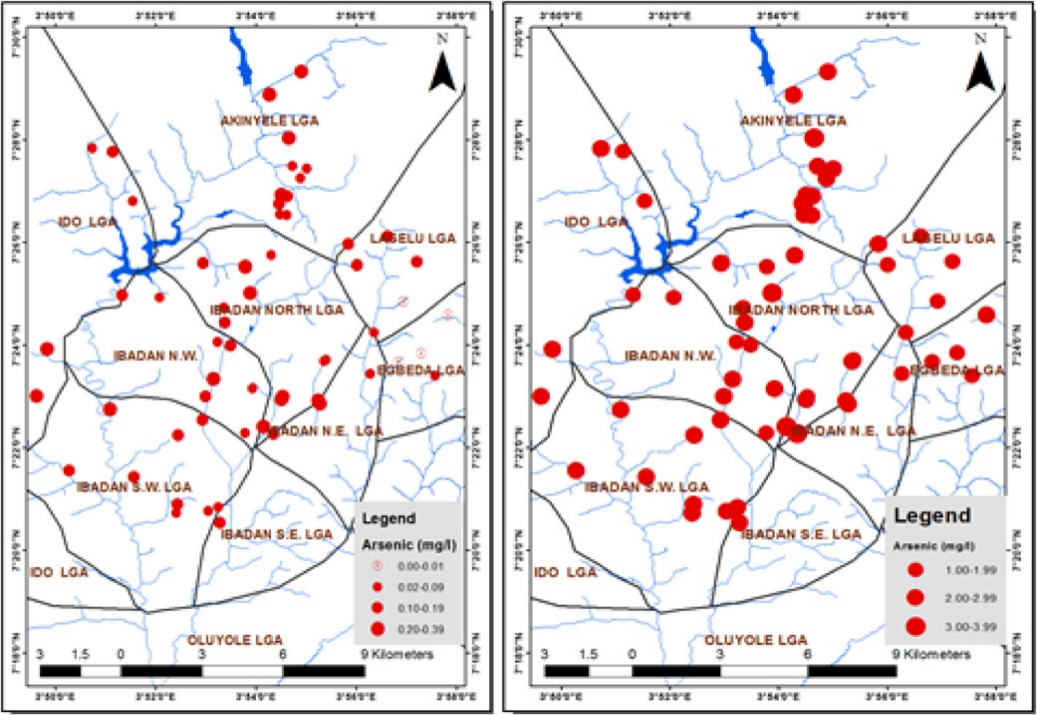
**Arsenic concentration (dry and rainy season) in groundwater.**

**Figure 4:**
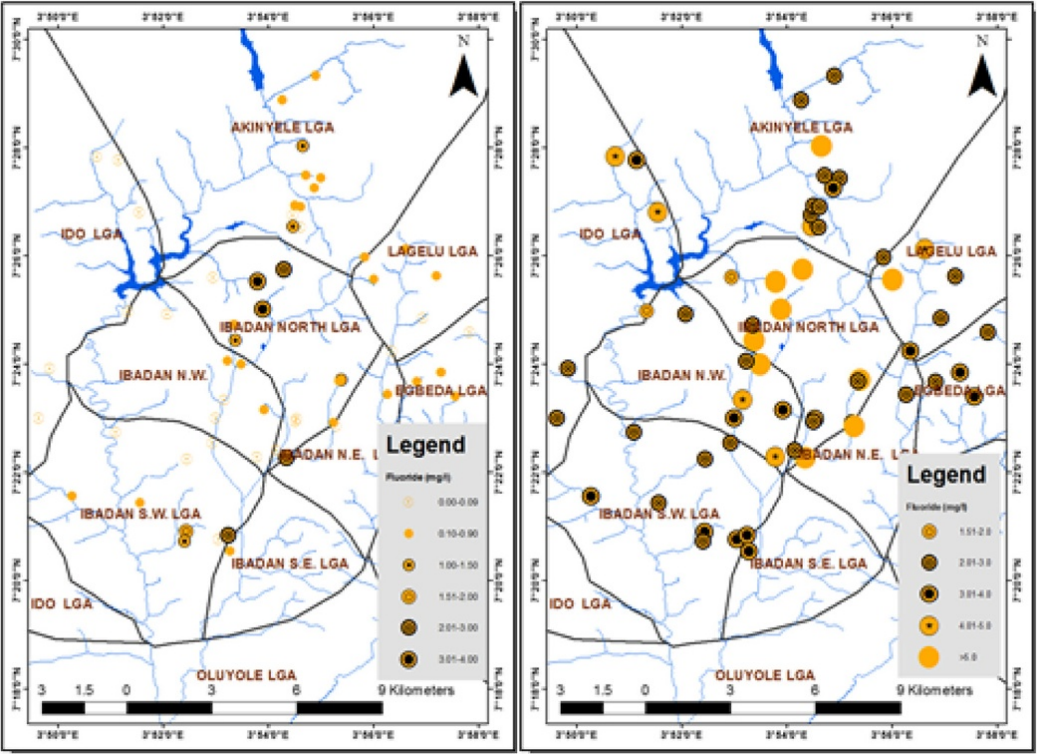
**Fluoride concentration (dry and rainy season) in groundwater.**

The results revealed high levels of concentration of arsenic, which exceeded the WHO ( 2011) recommended limit of 0.01 mg/l for drinking water in 98% of the dry season samples and 100% of the wet season samples. In the case of F., 13% of the dry season and 100% of the wet season samples exceeded the WHO ( 1984) recommended safe limit as shown in Figures 2, 3 and 4.

### Perception of groundwater quality within the study area

**Figure 5:**
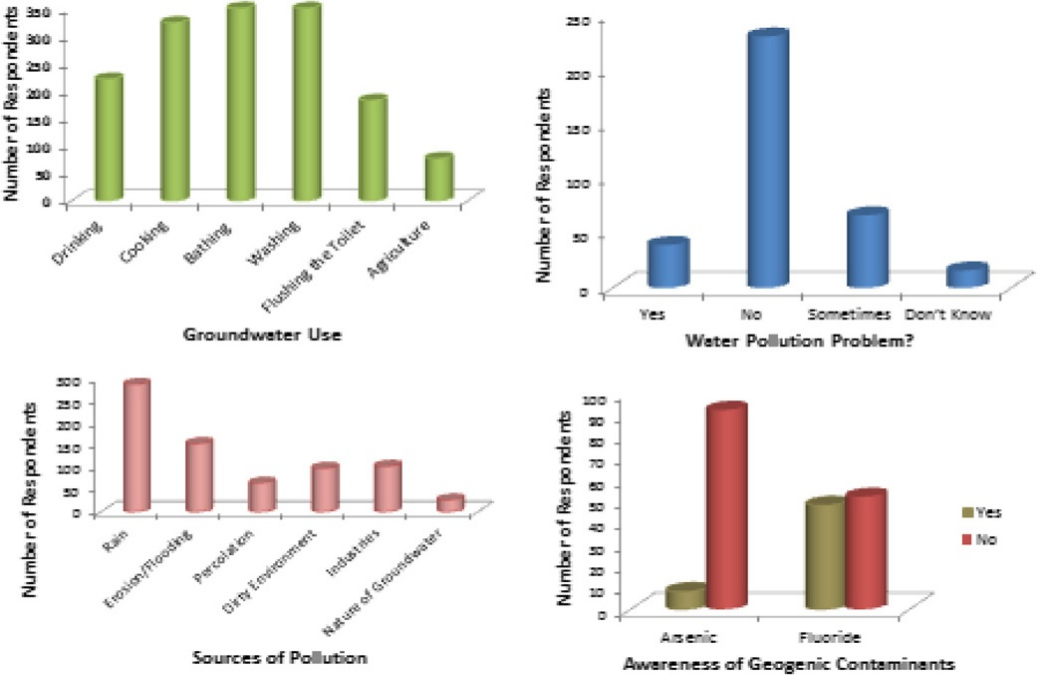
**Respondent views on groundwater contamination.**

Out of the 350 respondents to the questionnaires within the study area 32% were male while 68% were female, 22% had no formal education, 30% had basic or primary education, 39% had secondary education while 9% were graduates. Respondents use the water for drinking (63%), cooking (96%), bathing (100%), washing (100%), flushing the toilet (52%) and farming (22%). Among the respondents, 65% felt that their well water was unpolluted and fit for most domestic purposes, whereas 11% felt that their water was polluted, 19% indicated seasonal pollution while 5% did not know the status of their water supply. The respondents know that their water is polluted when there is a change in colour (25%), odour (53%), taste (37%) or when there are particles in the water (52%). Perceived sources of pollution include rainfall (82%), erosion/flooding (28%), percolation (21%), dirty environment (43%), industries (19%) and nature of water (24%) as shown in Figure 5. When asked if their well water had been tested for their physico-chemical properties, (85%) indicated that their well water had never been analyzed, while 15% claimed the water was analyzed when the wells were initially dug. Only 45% had heard about geogenic contamination, 8% had heard about arsenic and 48% fluoride. However, 71% will want their water tested for contaminants and 36% are willing to pay to have their wells tested. Furthermore, 92% will want their wells treated if contaminated by arsenic or fluoride and 47% are willing to pay for the treatment.

## Discussion

The highly elevated levels of concentration of arsenic and fluoride that were observed at the beginning of the rainy season as compared to the end of rainy season shows seasonal disparities in contaminant concentration. Arsenic and fluoride contamination of groundwater has been reported in many parts of the world. Elevated values of As. have been recorded in more than 10million tube wells used by about 30 to 40 million people in Bangladesh (British Geological Survey, Bangladesh Department of Public Health Engineering 2001; Wasserman, *et al.,*^2004^). Few studies have been carried out in Nigeria. Garba *et al*. ( 2010) reported mean arsenic concentration of 0.34 mg/l in drinking water from hand dug wells, boreholes and taps in Karaye Local Government area, Kano state, Northern Nigeria, Kayode *et al.* ( 2011) also reported high levels of arsenic contamination of drinking water from wells, boreholes and surface water sources in Ogun State (South West Nigeria). In these areas of elevated concentrations, groundwater is the main source of water for domestic purposes. The national population census revealed that within Ibadan, groundwater is the main source of water supply for 63% of households. This study also showed that 63% of respondents ingested the water indicating a high rate of exposure. As indicated above, high levels of As in groundwater have been known to cause a variety of health problems.

However, despite the health risks associated with these contaminants, the study revealed a complete lack of awareness of their presence in groundwater within the study area. For example, only 8% of respondents were aware that arsenic could contaminate groundwater while of the 48% that have heard of fluoride, most linked it with toothpaste. Without the benefit of chemical analysis, 65% of well owners had full faith in their water sources, most probably because of their low perception of water pollution. Most were of the view that if the well was ringed and covered then there was little or no risk of pollution of groundwater resources. They also supposed that the absence of colour, odour, taste and particles in the water meant the water was not polluted but clean.

Most of the wells within the study area as in most parts of Nigeria are privately owned, and there is no law making it mandatory for private owners to test the water from their wells. This may account for the low number of people that have had their wells tested. The study however revealed a willingness by respondents to have their well water tested. But most of the respondents were not willing to pay to have their water tested. Some believed that the government should be in charge of water quality problems and as such should test wells free of charge. In some countries, groundwater quality is monitored by the government. For example, groundwater quality in India is monitored by the Central Ground Water Board on a yearly basis (Central Groundwater Board 2010). Other countries such as Bangladesh that have the problem of geogenic contamination of groundwater also have networks of wells for groundwater quality monitoring.

## Conclusion

Groundwater is the most important source of freshwater for domestic use in Ibadan as well as in most cities in Nigeria. The study showed that most private well owners and users are not aware of contamination problems, particularly those of geogenic origin. The health implications of continued exposure to excessive levels of arsenic and fluoride in drinking water make the presence of geogenic contaminants and the lack of awareness of their presence a matter of grave concern. It is recommended that test of private wells and boreholes which tap into both deep and shallow aquifers should be carried out and remedial measures for the treatment of contaminated wells be effected within the study area.
